# Comparison of two rapid diagnostic tests during a large dengue virus serotype 3 outbreak in the Solomon Islands in 2013

**DOI:** 10.1371/journal.pone.0202304

**Published:** 2018-08-10

**Authors:** Li-Teh Liu, Tenneth Dalipanda, Rooney Jagilly, Ying-Hui Wang, Ping-Chang Lin, Ching-Yi Tsai, Wen-Ter Lai, Jih-Jin Tsai

**Affiliations:** 1 Department of Medical Laboratory Science and Biotechnology, College of Medicine and Life Science, Chung-Hwa University of Medical Technology, Tainan, Taiwan; 2 Center for Dengue Fever Control and Research, Kaohsiung Medical University, Kaohsiung, Taiwan; 3 Ministry of Health and Medical Services, Honiara, Solomon Islands; 4 National Referral Hospital, Honiara, Solomon Islands; 5 Tropical Medicine Center, Kaohsiung Medical University Hospital, Kaohsiung, Taiwan; 6 School of Medicine, College of Medicine, Kaohsiung Medical University, Kaohsiung, Taiwan; 7 Division of Cardiology, Department of Internal Medicine, Kaohsiung Medical University Hospital, Kaohsiung, Taiwan; 8 Division of Infectious Diseases, Department of Internal Medicine, Kaohsiung Medical University Hospital, Kaohsiung, Taiwan; National Yang-Ming University, TAIWAN

## Abstract

Dengue virus (DENV) infection causes various clinical presentations, including asymptomatic infection, dengue with or without warning signs and severe dengue. An early and accurate diagnosis of DENV infection during the first few days of illness supports clinical management and significantly reduces dengue-associated mortality and morbidity. However, it is very difficult to confirm DENV infection in endemic regions without qualified dengue diagnostic laboratories. In this study, we evaluated the performance of two commercially available rapid diagnostic tests (RDTs) using serum samples collected in the Solomon Islands during the 2013 DENV-3 outbreak. The sensitivity and specificity of the tests were calculated by comparing the results of DENV nonstructural protein 1 (NS1), IgM and IgG RDTs with those obtained by qRT-PCR. We also compared the results of the DENV IgM/IgG RDT with those obtained using an IgM/IgG capture enzyme-linked immune-sorbent assay (ELISA). The sensitivities of the SD and CTK NS1 RDTs were similar (90.9% and 92.6%), and the specificity of the SD NS1 RDT was significantly higher than that of the CTK NS1 RDT (100% versus 78.8%). The inclusion of IgM and IgG in the RDT did not significantly increase the sensitivity for DENV diagnosis. Compared with the SD IgM RDT, IgM capture ELISA had the same specificity but higher sensitivity. User-friendly RDTs remain the first choice and the most convenient tool in dengue endemic regions, where laboratory facilities and the corresponding infrastructure are lacking. Our study provided important and practical information for comparing the performance and validity of the different RDTs for rapid dengue detection.

## Introduction

Dengue is a mosquito-borne viral disease prevalent in the tropics and subtropics, where it causes significant morbidity and mortality. The distribution of dengue has increased greatly over the past 50 years, and this expansion is likely due to global warming and travel, which have increased the geographic expansion of the mosquito vectors. The disease is caused by infection with the dengue virus (DENV), an RNA virus consisting of a positive-sense RNA genome of approximately 11 kilobases. The DENV genome encodes three structural proteins (capsid, premembrane and envelope) and seven nonstructural proteins (NS1, NS2A/B, NS3, NS4A/B and NS5) [1]. DENV belongs to the genus *Flavivirus* and the family *Flaviviridae* and is classified into four serotypes (DENV-1 to DENV-4) based on serology. DENV is transmitted to humans by bites from infected *Aedes albopictus* and *Aedes aegypti* [2]. It has been estimated that 390 million DENV infections occur each year. DENV infection results in a wide spectrum of presentations, ranging from asymptomatic or mild illness to dengue fever (DF), dengue hemorrhagic fever (DHF) and dengue shock syndrome (DSS) [3], dengue with or without warning signs and severe dengue [4].

Although the methods for dengue diagnosis differ among countries and areas in the Western Pacific Region, a continuous and systemic national dengue surveillance program is organized by the WHO to monitor the dengue threat and its impact on health and the economy. The most recent official communication revealed that more than 200,000 cases have been consistently reported annually in the Western Pacific Region since 2007 [5]. The available evidence suggests that DENV has circulated in the Solomon Islands (SI) in the past [6]. However, with the exception of an outbreak in Honiara, the capital of the SI, in 1982 [7], information on dengue outbreaks or dengue disease in the SI was lacking [5,7] until a very recent report by the WHO describing the dengue outbreak that occurred from January to May, 2013 [8].

In 2009, Kaohsiung Medical University Hospital (KMUH) organized a medical team to provide medical services to people in the SI through the Taiwan Health Center (THC) located at the National Referral Hospital (NRH) in Honiara. In addition to malaria, we found evidence of dengue cases in a pilot study conducted during the years 2010–2011, which suggested that DENV might have been circulating in the SI, and the situation could be a public health concern. Thus, we initiated a prospective study in late 2012 to investigate the incidence and seroprevalence of dengue and its impact on the health of the people in the SI. The first dengue outbreak to occur in the SI in the past three decades was reported to have taken place from January to May in 2013. We collected 412 acute-stage sera samples from patients with dengue-like illness during the 2013 dengue outbreak. The sera were used to evaluate commercially available dengue NS1 and IgM/IgG rapid diagnostic tests (RDTs), the SD Dengue Duo, and CTK Dengue Ag and Dengue IgG/IgM Combo RDTs and DENV real-time quantitative RT-PCR (qRT-PCR) was used as the reference method for diagnosis [9]. We aimed to compare different RDTs for rapid dengue detection in the SI, where laboratory facilities and the corresponding infrastructure are lacking.

## Materials and methods

### Patients and sample collection

This study was approved by the Institutional Review Board of KMUH (KMUHIRB-20120080) and the National Health Research & Ethics Committee of the Ministry of Health and Medical Services (MHMS) of the SI (HRC 13/16). During the 2013 dengue outbreak, acute-phase serum samples were collected from 412 patients with dengue-like illness at the NRH in Honiara, Guadalcanal Province, SI, by team members of the Taiwan Health Center. A confirmed case of DENV infection was defined as presenting with dengue-like illness and having a positive real-time qRT-PCR result. Patients with dengue-like illness and negative real-time qRT-PCR results were defined as having other febrile illness (OFI). Sera collected between days 0 and 6 after the onset of fever or symptoms were regarded as acute-phase samples. The methods used in this study included the SD Dengue Duo, CTK Dengue Ag and Dengue IgG/IgM Combo RDTs, dengue real-time qRT-PCR [10], and anti-DENV IgM and IgG capture ELISA [11]. All the tests were performed at the Tropical Medicine Center (TMC) at KMUH in Taiwan. The TMC is an ISO 15189 certified dengue diagnostic laboratory authorized by the Taiwan Centers for Disease Control (CDC), the Taiwan Accreditation Foundation and the International Laboratory Accreditation Cooperation Laboratory Combined Mutual Recognition Arrangement Mark.

### Preparation of DENV

Four serotypes of DENV, including four prototype strains (DENV-1, US/Hawaii/1994; DENV-2, New Guinea C; DENV-3, DN8700829A; and DENV-4, DN9000475A), were propagated in C6/36 cells, and viral titers were determined by plaque assay using BHK21 cells. The DENV-infected supernatants were used in the anti-DENV IgM/IgG capture ELISAs and as a positive control in the real-time qRT-PCR assay.

### Viral RNA isolation and real-time qRT-PCR

Viral RNAs were extracted from 200 μl of the cell culture supernatants or serum samples using the PureLink^®^ Viral RNA Mini Kit (Life Technologies; USA) according to the manufacturer’s instructions and stored at -80°C. Real-time qRT-PCR was performed using a Mx3000P quantitative PCR system (Agilent/Stratagene, USA). The samples were assayed in 25-μl reaction mixtures containing 5 μl of the sample RNA and 0.2 μM forward and reverse primers using the Brilliant II SYBR Green QRT-PCR Low ROX Master Mix and RT/RNase Block Enzyme Mixture (Agilent/Stratagene, USA). The primer sequences applied for detecting and typing the dengue viruses were published previously [10]. The thermal profile used for the one-step SYBR Green-based real-time qRT-PCR assay and the melting curve analysis were based on previously described information with minor modifications [10], and amplification plots and Tm values were routinely analyzed to verify the specificity of the amplicons.

### Dengue NS1 antigen and anti-DENV IgM/IgG detection using RDTs

SD Dengue Duo (Standard Diagnostics, Inc., Korea) and Dengue Ag Rapid Test-Cassette (CTK Biotech, Inc., USA) were used to detect DENV NS1 antigens in serum samples. SD Dengue Duo and Dengue IgG/IgM Combo Rapid Test cassettes were used for the rapid detection of anti-DENV IgG and IgM in serum samples. The CTK products were provided by the manufacturer. All RDTs were performed following the manufacturer’s instructions.

### Capture anti-DENV IgM and IgG ELISA

Anti-DENV IgM and IgG ELISAs were performed as described previously [11] with some modifications. In brief, antihuman IgM- or antihuman IgG-coated microplates (Taiwan Advance Bio-Pharmaceutical, Inc, Taiwan) were incubated with 100 μl of the serum samples, the positive controls, which included DENV IgM- or IgG-positive serum, or the negative controls, which included DENV IgM- or IgG-negative serum, at 1:100 dilution in casein buffer containing 2.5% normal rabbit serum at 37°C for 30 min. The microplates were then washed four times with 350 μl of PBST (1X phosphate-buffered saline with 0.05% Tween 20). After washing, the microplates were incubated with 100 μl of a mixture containing pooled ultraviolet (UV)-inactivated DENV 1–4 virus antigens combined with anti-flavivirus envelope (E) protein monoclonal antibody (Yao-Hong Biotechnology, Taiwan) diluted 1:1000 in dilution buffer (1X phosphate-buffered saline, 0.1% Tween 20, 1% bovine serum albumin, 5% normal rabbit serum, and 0.02% sodium azide, pH 7.2~7.4) at 37°C for 30 min. The microplates were then washed four times with 350 μl of PBST. After washing, 100 μl of goat anti-mouse IgG-AP conjugates at 1:1000 dilution (Jackson ImmunoResearch, USA) was added to the microplates, and the microplates were incubated at 37°C for 30 mins. The microplates were then washed and incubated with 100 μl of p-nitrophenyl-phosphate (Millipore, USA) at 37°C. After incubation for 40 and 70 min, the optical density was determined at a wavelength of 405 nm with a reference wavelength of 630 nm using a μQuant™ Model MQX200 Microplate Spectrophotometer (BioTek® Instruments, Inc., USA). A positive sample was defined as a sample with a test absorbance ≥0.5 and a ratio of the positive to negative controls ≥2.0, and a negative sample was defined a sample having a test absorbance <0.5. Most importantly, primary or secondary dengue virus infection was defined by the ratio of the IgM to IgG readings (≥1.2 or <1.2, respectively) [12].

### Statistical analysis

All statistical analyses were performed using SPSS (version 14.0, SPSS, Chicago, IL, USA). The kappa coefficient values were determined to assess the agreement of the mean sensitivity, specificity, positive predictive value (PPV), and negative predictive value (NPV) of each test with those of the reference standard.

## Results

### Characteristics of the study participants

The characteristics of the participants with dengue-like illness enrolled in this study (n = 412) are shown in Table 1. The confirmed DF cases (n = 242) were defined based on the WHO case definition and positive results in the DENV real-time qRT-PCR assay [4]. Patients with negative real-time qRT-PCR results (n = 170) were classified as having other febrile illness (OFI). The median duration of illness prior to sample collection was 2 days for both groups. The infecting DENV serotype in all DF cases were determined by serotype-specific RT-PCR and by sequencing ten randomly selected cases. The results revealed that all positive real-time qRT-PCR samples were DENV-3. Based on the ratio of the IgM to IgG readings, 89.3% of the confirmed dengue cases were primary infections (n = 216), and 5.8% were secondary infections (n = 14) [12].

**Table 1:** Characteristics of the study cohort in the Solomon Islands.

Variable^a^	Confirmed dengue^b^	Other febrile illness
	n = 242	n = 170
Male sex	112 (50.5%)	48 (45.7%)
Age	26 (3–67)	27 (1–102)
Day of illness	2 (0–6)	2 (0–6)
Infecting serotype and serological status		
DENV-3	242 (100%)	
Primary	216 (89.3%)	
Secondary	14 (5.8%)	
Indeterminate^c^	12 (5%)	

^a^ Presented as the numbers (%) except age and day, which are shown as the medians (ranges).

^b^ Confirmed dengue was based on positive DENV qRT-PCR results.

^c^ The sample volumes for 12 cases were insufficient for the IgM or IgG analysis.

### Sensitivity and specificity of the NS1 tests

Table 2 summarizes the sensitivity and specificity of different tests compared with those of qRT-PCR, which was used as the reference. No significant difference in the sensitivity of the SD NS1 and CTK NS1 RDTs for the diagnosis of acute dengue was observed (SD NS1 90.9% *vs* CTK NS1 92.6%, *P =* 0.5). The specificity of the SD NS1 RDT was 100%, which was significantly higher than that of the CTK NS1 RDT (100% *vs* 78.8%, *P<0*.*001*). The inclusion of IgM alone or in combination with IgG in the SD RDTs did not significantly increase either the sensitivity (93.0% *vs* 90.9%, *P =* 0.4) or the specificity of the RDTs compared with that obtained with NS1 alone. In the CTK RDTs, the inclusion of IgM did not significantly increase the sensitivity (94.6% *vs* 92.6%, *P =* 0.35), and the inclusion of IgM or IgG increased the sensitivity (98.3% *vs* 92.6%, *P =* 0.002), but the inclusion of IgM (62.4% *vs 78*.*8*%, *P =* 0.001) or IgM/IgG (24.1% *vs 78*.*8*%, *P*<0.001) greatly reduced the specificity compared with that obtained with NS1 alone. The comparison of the sensitivity of the SD NS1 RDT plus IgM/IgG with that of the SD NS1 RDT plus the time-consuming capture IgM/IgG ELISA did not reveal a significant difference (93% *vs* 95.5%, *P* = 0.3).

**Table 2:** Sensitivity and specificity of each assay based compared with those of the reference qRT-PCR Test.

Assay parameter	Total patients (n)	Acute dengue cases^a^ (n)	Number of positive results (n)	Sensitivity^b^	Specificity^c^	Kappa value^d^	PPV^e^	NPV ^f^
SD NS1	412	242	220	90.9% (220/242)	100% (170/170)	0.8919 (0.8482–0.9356)	100% (220/220)	88.5% (170/192)
SD NS1 or IgM	412	242	225	93% (225/242)	100% (170/170)	0.9161 (0.8772–0.955)	100% (225/225)	90.9% (170/187)
SD NS1 or IgM or IgG	412	242	225	93% (225/242)	100% (170/170)	0.9161 (0.8772–0.955)	100% (225/225)	90.9% (170/187)
CTK NS1	412	242	224	92.6% (224/242)	78.8% (134/170)	0.7253 (0.6574–0.7932)	86.2% (224/260)	88.2% (134/152)
CTK NS1 or IgM	412	242	229	94.6% (229/242)	62.4% (106/170)	0.5964 (0.5184–0.6744)	78.2% (229/293)	89.1% (106/119)
CTK NS1 or IgM or IgG	412	242	238	98.3% (238/242)	24.1% (41/170)	0.2522 (0.1788–0.3256)	64.9% (238/367)	91.1% (41/45)
SD NS1 or IgM ELISA	412	242	229	94.6% (229/242)	100% (170/170)	0.9356 (0.9012–0.97)	100% (229/229)	92.9% (170/183)
SD NS1 or IgM/IgG ELISA	412	242	231	95.5% (231/242)	100% (170/170)	0.9454 (0.9136–0.9772)	100% (231/231)	93.9% (170/181)

^a^ Based on a positive DENV qRT-PCR result.

^b^ The sensitivity is presented as a % (number of positive results with NS1/number of positive qRT-PCR results).

^c^ The specificity is based on 170 samples from patients with other febrile illness (negative qRT-PCR results).

^d^ The parentheses indicate the 95% confidence intervals.

^e^ Positive predictive value (PPV).

^f^ Negative predictive value (NPV).

### Sensitivity of NS1, IgM and IgG RDTs and sampling time

The sensitivities of the SD and CTK NS1 RDTs did not show significant differences between specimens collected at different time periods (Table 3). Our results agreed with those reported by Tricou et al. [13]. However, other studies suggested that the DENV NS1 RDT showed higher sensitivity with samples collected within 3 days post-symptom onset (PSO) than those collected after 3 days PSO [14–17]. Overall, the inclusion of IgM or IgM/IgG in both the SD and CTK NS1 RDTs using samples collected within 3 days PSO increased the sensitivity of these tests for the diagnosis of acute dengue, but the differences were not significant. For samples collected after 3 days PSO, the inclusion of IgM or IgM/IgG did not significantly increase the sensitivity of the SD NS1 RDT or that of the CTK NS1 RDT.

**Table 3:** Sensitivity of the dengue RDTs for samples collected at different time periods.

		SD NS1	SD NS1 or IgM	SD NS1 or IgM or IgG	CTK NS1	CTK NS1 or IgM	CTK NS1 or IgM or IgG
Status ^a^	Total (n)			% Sensitivity			
Collected ≤3 days PSO	88	95.5% (84/88)	96.6% (85/88)	96.6% (85/88)	94.3% (83/88)	95.5% (84/88)	97.7% (86/88)
Collected >3 days PSO	13	92.3% (12/13)	92.3% (12/13)	92.3% (12/13)	92.3% (12/13)	92.3% (12/13)	100% (13/13)
P value^b^		0.505	0.429	0.429	0.572	0.505	1.000

^a^ PSO: post-symptom onset

^b^ Fisher's exact test

### NS1 detection in primary and secondary DENV infections

The NS1 detection rates in patients with primary DENV infection obtained using both SD and CTK RDTs did not show significant differences (Table 4). For the primary DENV infection panel, the inclusion of IgM did not significantly increase the sensitivity of the SD (93.1% *vs* 91.2%, *P* = 0.5) and CTK (94% *vs* 92.1%, *P* = 0.4) RDTs. The inclusion of IgM or IgG significantly increased the sensitivity of the CTK RDT (98.1% *vs* 92.1%, *P* = 0.04) but not that of the SD RDT (93.1% *vs* 91.2%, *P* = 0.5). In the case of secondary DENV infections, the sensitivity of the CTK NS1 RDTs was higher than that of the SD NS1 RDT, but the difference was not significant (92.9% *vs* 78.6%, *P* = 0.6). Inclusion of the IgM and IgM/IgG parameters in the SD (85.7% *vs* 78.6%, *P* = 1) and CTK (100.0% *vs* 92.9%, *P* = 1) RDTs did not significantly increase the sensitivity of these tests for the diagnosis of patients with secondary DENV infection compared with that obtained with NS1 alone. The NS1 detection rates in patients with secondary infections obtained using the SD NS1 RDT were lower than those in patients with primary infections (78.6% *vs* 91.2%), but this difference was not significant (*P* = 0.138). Similarly, no significant difference in the NS1 detection rates obtained with the CTK NS1 RDT was obtained between primary and secondary DENV infections (92.1% *vs* 92.9%, *P* = 1). The sensitivity of NS1 RDT was affected by the viremia levels, as demonstrated by the comparison of patients with primary and secondary DENV infections (Ct for primary infection: 25.46 (range 14.66–36.58, n = 216) *vs* secondary infection: 27.7 (range 23.19–35.23, n = 14), *P* = 0.039).

**Table 4:** Sensitivity of dengue RDTs for patients with primary and secondary infections.

		SD NS1	SD NS1 or IgM	SD NS1 or IgM or IgG	CTK NS1	CTK NS1 or IgM	CTK NS1 or IgM or IgG	Sampling day ^c^	Ct ^d^
Status	Total (n)	Sensitivity						Median (range)	
Primary infection	216	91.2% (197/216)	93.1% (201/216)	93.1% (201/216)	92.1% ^b^ (199/216)	94% (203/216)	98.1% ^b^ (212/216)	2 (0–5)	25.46 (14.66–36.58)
Secondary infection	14	78.6% (11/14)	85.7% (12/14)	85.7% (12/14)	92.9% (13/14)	100% (14/14)	100% (14/14)	4 (2–6)	27.7 (23.19–35.23)
P value^a^		0.138	0.277	0.277	1.000	1.000	1.000	0.001	0.039

^a^ Fisher's exact test was used to compare the sensitivity between primary and secondary infections.

^b^ Fisher's exact test was used to compare the sensitivity between CTK NS1 and CTK NS1 or IgM/IgG; P = 0.04.

^c^ Sampling day; days post-symptom onset.

^d^ Mean Ct values (range) of dengue qRT-PCR assay.

### Effects of anti-DENV IgG on the assay performance of NS1 RDTs

The sensitivity of the NS1 RDT for dengue diagnosis is likely to be affected by serological factors such as DENV-reactive IgG [13]. Thus, we analyzed the effect of DENV-reactive IgG on the assay performance of NS1 RDTs. The presence of anti-DENV IgG was assessed by ELISA [11]. Of the 242 DENV qRT-PCR-positive cases, 15 and 213 were positive and negative for IgG, respectively (Table 5). The results suggested that the presence of anti-DENV IgG decreased the sensitivity of the SD NS1 RDT, but the differences were not statistically significant (80% *vs* 91.1%, *P* = 0.17). The sensitivity of the CTK NS1 RDTs was not decreased in the presence of anti-DENV IgG. Thirteen of the 15 IgG-positive cases were cases of secondary infection. These results suggested that the presence of DENV IgG in the test sample might hinder the detection of DENV NS1 using the SD NS1 RDT.

**Table 5:** Sensitivity of SD and CTK NS1 RDTs in the presence or absence of DENV IgG.

	SD NS1 1		CTK NS1 1	
DENV-IgG ELISA ^a^	IgG+	IgG-	IgG+	IgG-
Confirmed acute dengue cases (n)	15	213	15	213
Sensitivity^b^	80% (12/15)	91.1% (194/213)	93.3% (14/15)	92% (196/213)
P value^c^	0.165		1.000	

^a^ DENV-IgG ELISA was performed with 228 dengue qRT-PCR confirmed samples.

^b^ The sensitivity is presented as a % (number of positive results with NS1/number of positive qRT-PCR results).

^c^ Fisher’s exact test

### Comparison of IgM/IgG RDT and IgM/IgG ELISA

The above results (Table 2) suggest that the performance of the IgM and IgG RDTs is as good as that of the time-consuming capture IgM and IgG ELISA. To understand the performance of the IgM/IgG RDTs and the capture IgM/IgG ELISA for dengue diagnosis, we compared the IgM and IgG results based on the reference method, dengue qRT-PCR (Table 6). The sensitivity of the SD IgM RDT was significantly lower than those of the CTK IgM RDT and IgM ELISA (9% vs 46.5% and 42.2% respectively, *P*<0.001). The specificity of the CTK IgM RDT was significantly lower than those of the SD IgM RDT and IgM ELISA (75.9% vs 100% and 100%, *P*<0.001). In the case of IgG, the sensitivity of the CTK IgG RDT was significantly higher than those of the SD IgG RDT and IgG ELISA (*P*<0.001). However, the specificity of the CTK IgG RDT was significantly lower than those of the SD IgG RDT and IgG ELISA (*P*<0.001). The comparison between the IgM and IgG results of the RDTs and the capture IgM and IgG ELISA results suggested that the consistency was poor (IgM RDT *vs* IgM ELISA, kappa value = 0.0029 and 0.0154 for SD and CTK RDTs, respectively; IgG RDT *vs* IgG ELISA, kappa value = 0.215 and 0.0502 for SD and CTK RDTs, respectively).

**Table 6:** Comparison of IgM/IgG RDT and IgM/IgG ELISA using dengue qRT-PCR as the reference.

	IgM		IgG	
Method	Sensitivity^a^	Specificity^b^	Sensitivity^a^	Specificity^b^
SD RDT	9% (21/230)	100% (170/170)	6.6% (15/228)	100% (170/170)
CTK RDT	46.5% (107/230)	75.9% (129/170)	73.2% (167/228)	36.5% (62/170)
IgM ELISA	42.2% (97/230)	100% (170/170)	-	-
IgG ELISA	-	-	6.6% (15/228)	100% (170/170)

^a^ The sensitivity is presented as a % (number of positive IgM or IgG results/number of positive qRT-PCR results).

^b^ The specificity is based on 170 samples from patients with other febrile illness (negative qRT-PCR results).

We subsequently compared the results of the IgM/IgG RDT using capture IgM and IgG ELISA as the reference method (Table 7). Compared with that of the CTK IgM test, the sensitivity of the SD IgM test was lower (P<0.001), and the specificity was higher (P<0.001). The sensitivity of the SD IgM RDT was notably lower than that claimed by the manufacturer (day 1–3: 21%, day 4–6: 48%); data for the CTK IgM RDT were not available. In the case of IgG, the sensitivity and specificity of the SD RDT were lower (P<0.001) and higher (P<0.001) than those of the CTK IgG RDT. The sensitivity of the SD IgG RDT was similar to that claimed by the manufacturer (day 1–3: 4%, day 4–6: 22%); data for the CTK IgG RDT were not available. The extremely lower sensitivity of the SD IgM RDT compared with that of the IgM ELISA might not be due to the temperature factor in the SI because the SD RDT was recently reported to remain stable even after long-term storage at high or fluctuating temperatures [18].

**Table 7:** Performance of the IgM/IgG RDT compared with that of dengue IgM/IgG ELISA as the reference.

	IgM			IgG		
Method	Sensitivity	Specificity	Kappa value	Sensitivity	Specificity	Kappa value
SD RDT	9.3% (9/97)	96% (291/303)	0.0725 (0–0.1558)	26.7% (4/15)	97.1% (372/383)	0.2379 (0.0247–0.4511)
CTK RDT	47.4% (46/97)	66.3% (201/303)	0.1167 (0.0206–0.2128)	100% (15/15)	32.1% (123/383)	0.0344 (0.0164–0.0524)
P value^a^	<0.001	<0.001		<0.001	<0.001	

^a^Fisher's exact test

## Discussion

In this study, we investigated the sensitivity and specificity of two commercially available lateral flow rapid tests for the detection of dengue NS1 antigen and IgM/IgG in acute-phase sera collected from patients with suspected DF during the DF outbreak from January to July in 2013 which is the largest one recorded over the past three decades in the SI [7,19]. The results showed that the two RDTs have similar sensitivities (90.9% *vs* 92.6%) compared with the DENV real-time qRT-PCR test, which was used as the reference. However, the specificity of SD NS1 RDT was notably better than that of the CTK NS1 RDT (100% *vs* 78.8%).

The inclusion of IgM in both RDTs slightly increased their sensitivity but reduced the specificity of the CTK RDT (62.4% *vs* 78.8%). The inclusion of IgG further improved the sensitivity of both RDTs but further reduced the specificity of the CTK RDT (24.1% *vs* 78.8%). In contrast, the inclusion of IgM and IgG slightly increased the sensitivity of the SD NS1 RDT but did not alter the specificity. The inclusion of anti-DENV IgM/IgG capture ELISA results in the SD NS1 RDT did not significantly increase the sensitivity of the test compared with that of the SD NS1 RDT including IgM/IgG (95.5% *vs* 93%).

We further analyzed whether the sensitivity of the SD and CTK NS1 RDTs was affected by the sampling time and found no difference in sensitivity between samples collected within and after 3 days PSO onset. Our results were in agreement with those reported by Tricou et al. [13]. However, other studies suggested that the DENV NS1 RDT showed higher sensitivity with samples collected within 3 days PSO than with samples collected after 3 days PSO [14–17]. The SD NS1 RDT shows higher sensitivity with primary DENV infection samples than with secondary infection samples, although the difference was not significant, likely due to the small sample size of secondary infection. In contrast, the CTK NS1 RDT showed similar sensitivity in both types of infection. The presence of IgG in a sample, as determined by IgG ELISA, decreased the sensitivity of the SD NS1 RDT but not that of the CTK NS1 RDT. These results obtained for the SD NS1 RDT were similar to the results reported by Tricou et al. [13].

Previous studies have found different sensitivities for the SD NS1 RDT, ranging from 44.4% to 87% [13,20–23]. The sensitivity of the CTK NS1 RDT reported by a previous study was 40% [24], and it has been reported that the RDTs show higher sensitivity with primary infection than with secondary infection samples [13,24–26]. The high sensitivity of both NS1 RDTs investigated in our study might have been obtained because most of our patients had primary infections (89.3%). In addition, we cannot evaluate the performance of the SD and CTK RDTs between the different DENV serotypes because all the samples included in the current study were obtained from patients with DENV-3 infections.

In recent studies, the sensitivity of DENV IgM capture ELISA for specimens collected within 6 days PSO ranged from 62.9% to 98% [24,26]. It appears that the sensitivity of our IgM capture ELISA (42.2%, 97/230) compared with that of qRT-PCR as the reference method was lower than the average, which might be due to the time of sampling (median was 2 days PSO in our study) because the use of specimens collected within 3 days PSO might decrease the sensitivity to 44–45.1% [27,28]. The following sensitivities have been reported for the DENV IgM RDTs: 89% [24] and 60.5% [26] for the SD IgM RDT and 46% [24] for the CTK IgM RDT [24,26]. It appears that the sensitivity of the CTK IgM RDT was reasonable and comparable to that of the IgM capture ELISA (46.5% vs 42.2%) using qRT-PCR as the reference method. The sensitivity of the CTK IgM RDT obtained using IgM capture ELISA as the reference method was 47.4%. However, the specificity of the SD IgM RDT was better than that of the CTK IgM RDT. It should be noted that poor consistency was obtained between the IgM RDT and IgM ELISA. The sensitivity of the SD IgM RDT obtained using qRT-PCR and IgM capture ELISA as the reference methods was only 9% (21/230) and 9.3% (9/97), respectively (Tables 6 and 7). In addition, the patients included in this study were all negative for malaria (based on malaria RDT or microscopy parasite smear); notably, false-positive IgM results have been reported in patients with malaria [29].

Serotype-specific RT-PCR confirmed that the outbreak was 100% DENV-3 (n = 242). Our serotyping result was in agreement with that found in a recent study conducted by Nogareda et al. [8]. Sequencing of the core genes and a phylogenetic analysis suggested that the DENV-3 strain likely originated from Indonesia, which is consistent with the findings reported by Cao-Lormeau et al. [30]. This DENV-3 outbreak was predicted by a previous study, which suggested that DENV-3 might reappear in the Pacific island states in approximately 2012 [31]. Nogareda et al. used the SD Dengue Duo to test sera collected from suspected DF cases and reported a positive rate of 39% (1220/3141); the positive rate obtained in our study was 54.6% (225/412) [8]. This difference might be due to the different sampling times used in these two studies. The previous study did not compare the results obtained with the SD NS1/IgM RDT with those obtained with RT-PCR and IgM capture ELISA. Furthermore, our results suggest that 5.8% of confirmed dengue patients had secondary DENV infections (14 cases), whereas the study conducted by Nogareda et al. did not identify secondary DENV infections. Our observations suggested that DENV was circulating in the SI in the past.

## Conclusions

In our study, we evaluated the sensitivity and specificity of two commercially available dengue NS1 and IgM/IgG RDTs using acute-phase sera collected in the SI during the 2013 DENV-3 outbreak. Taken together, our results suggested that the sensitivities of the SD and CTK NS1 RDTs were similar (~90%), whereas the specificity of the SD NS1 RDT obtained using DENV qRT-PCR as the reference method was higher than that of the CTK NS1 RDT. The inclusion of IgM/IgG RDT did not significantly increase its diagnostic sensitivity of DENV infection. Although there is currently no dengue RDT that can provide a definitive diagnosis of DENV infection and other diagnostic methods or algorithms have been developed for the detection of DENV infection [32,33], user-friendly RDTs remain the first choice and the most convenient tool in dengue endemic regions where laboratory facility and resources and the corresponding infrastructure are still lacking. Our study provided important and practical information for comparing the performance and validity of different RDTs for rapid dengue detection. We suggest that the DENV NS1 RDT has the ability to differentiate patients that are truly infected with DENV from patients with other febrile illness and can improve patient management in resource-limited areas, such as the Solomon Islands, where DENV has circulated in the past and secondary DENV infections might cause severe outcomes in the future.
